# Self-management of long COVID symptoms with over-the-counter medicines and other non-prescribed therapies: a cross-sectional survey

**DOI:** 10.1186/s12889-026-28017-5

**Published:** 2026-07-02

**Authors:** Naijie Guan, Grace Turner, Richard Hotham, Daniel Lange, Kirsty R. Brown, Christel McMullan, Sarah E. Hughes, Olalekan Lee Aiyegbusi, Karen Matthews, Louise Jackson, Asma Yahyouche, Yvonne Alder, Felicity Jeyes, Lewis Buckland, Amy Chong, David Stanton, Melanie Calvert, Shamil Haroon

**Affiliations:** 1 Department of Applied Health Sciences, University of Birmingham, Birmingham, UK; 2 School of Sport, Exercise and Rehabilitation Sciences, University of Birmingham, Birmingham, UK; 3 Centre for Patient Reported Outcomes Research, University of Birmingham, Birmingham, UK; 4 NIHR Birmingham Biomedical Research Centre, University of Birmingham, Birmingham, UK; 5 NIHR Birmingham-Oxford Blood and Transplant Research Unit (BTRU) in Precision Transplant and Cellular Therapeutics, University of Birmingham, Birmingham, UK; 6 National Institute for Health and Care Research (NIHR) Applied Research Collaboration West Midlands, Birmingham, UK; 7 Birmingham Health Partners Centre for Regulatory Science and Innovation, University of Birmingham, Birmingham, UK; 8 Long Covid SOS, Charity Registered in England & Wales, 11 A Westland Road, Faringdon, Oxfordshire SN7 7EX UK; 9 Institute of Clinical Sciences, School of Pharmacy, University of Birmingham, Birmingham, UK

**Keywords:** Long COVID, Self-management, Over-the-counter medicines, Alternative therapies

## Abstract

**Background:**

The high prevalence of long COVID globally necessitates investigation into its self-management, especially given the absence of definitive and effective treatments and uneven access to healthcare services.

**Methods:**

This study surveyed the use of over-the-counter (OTC) medicines, supplements, remedies, and other non-prescription therapies for managing long COVID symptoms in the UK. It aimed to identify the range of treatments used for self-management, explore the sources of these treatments, factors influencing treatment choices, and associated out-of-pocket expenses. A cross-sectional electronic survey was provided to individuals experiencing long COVID. It included questions on the use of OTC medications, supplements, and other therapies, where they were sourced, decision-making influences, and financial costs. Descriptive statistics and thematic analysis were applied to analyse the data.

**Results:**

Among the 193 surveyed participants, significant use of vitamins, minerals, and herbal treatments (88.8%), and analgesics (73.6%) was reported, with 42% exceeding recommended dosages. Some participants sought relief through alternative therapies such as physiotherapy and acupuncture, often incurring significant personal expenses. Choices about self-management were influenced by medical professionals, family, friends, and online sources, including support groups and social media.

**Conclusions:**

People with long COVID may access a wide range of OTC medicines, dietary supplements, herbal remedies, and non-pharmacological therapies to self-manage symptoms. Healthcare providers should be aware of the use of non-prescribed therapies among long COVID sufferers and consider these in their treatment plans. Public health policies should focus on providing accurate information and guidance for patients self-managing long COVID symptoms.

**Supplementary Information:**

The online version contains supplementary material available at https://doi.org/10.1186/s12889-026-28017-5.

## Background

Long COVID, also known as post-acute sequelae of SARS-CoV-2 infection, has emerged as a significant concern in the aftermath of the COVID-19 pandemic. While the acute phase of COVID-19 may last a few weeks, a considerable number of patients continue to report a wide array of symptoms beyond this period. In some individuals, these symptoms persist far longer, and if they are present beyond 12 weeks post-infection, they are defined as having long COVID [1]. These symptoms include but are not limited to fatigue, breathlessness, and neurocognitive challenges [2–5]. The prevalence of long COVID in the UK and worldwide is high. As of March 2024, over 2 million people were estimated to be experiencing self-reported long COVID in the UK alone [6]. Globally, an estimated 6.2% of individuals who survived the acute phase of SARS-CoV-2 infection developed at least one long COVID symptom three months after the infection [7]. The prevalence and associated burden of long COVID have steadily increased throughout the pandemic. Among individuals self-reporting long COVID, 79% reported that their symptoms adversely affected day-to-day activities, quality of life, work capability, and well-being [8]. These impacts highlight the management of long COVID symptoms as an important area of research and public health consideration.

There are currently no specific and definitive treatments for long COVID. The underlying pathophysiology of long COVID symptoms is still unclear, although the existing evidence proposes potential mechanisms for long COVID pathogenesis, including immune dysregulation, microbiota disruption, autoimmunity, clotting and endothelial abnormality, and dysfunctional neurological signalling [9, 10]. Current long COVID management is therefore largely symptom-based and should be tailored to individual needs [11]. Specialist post-COVID services have been set up in several parts of the UK, including the National Health Service (NHS) post-COVID clinical services across England [12] and Wales. These multidisciplinary services involve physical, cognitive, and psychological examinations, diagnostic testing, management, or appropriate referral to post-COVID rehabilitation, therapy, and other assistance. However, their accessibility has been uneven and highly variable in healthcare provision, and many people continue to experience unmet needs [13].

In the absence of definitive curative treatments and with ongoing unmet needs in specialist services, self-management has become an important component of long COVID care [1, 11, 14, 15]. People may self-manage their long COVID symptoms using a range of approaches, such as over-the-counter (OTC) medicines, dietary supplements, herbal remedies, as well as complementary and alternative therapies. Prior studies conducted during the COVID-19 pandemic has reported self-medication for acute COVID-19 symptoms, including the use of analgesics, vitamins, antibiotics, traditional medicines and other medicines [15–17]. This trend towards self-prescription has been observed similarly among people with long COVID [17, 18]. Non-pharmacological approaches, including telerehabilitation, neuromodulation and exercise-based interventions, have also been reported for managing persistent COVID-19-related symptoms [19]. However, existing research has largely focused on the self-management of acute COVID-19 symptoms, while little is known about the range of OTC medicines and other non-prescribed therapies used by people living with long COVID.

While some self-management approaches may offer symptomatic relief and a sense of control for people with long COVID, they are not without potential risks [20]. The use of non-prescribed medicines, supplements, and remedies may raise concerns about inappropriate dosing, prolonged or off-label use, drug–drug interactions, and adverse effects [18, 20]. Additionally, individuals may also incur considerable out-of-pocket expenditure on products or therapies with limited or uncertain evidence of effectiveness. These concerns are particularly relevant for long COVID, given the diversity and persistence of symptoms and the potential for individuals to use multiple products or therapies simultaneously.

Given the lack of established curative treatments for long COVID and the potential risks and costs associated with self-management among affected people, it is important to understand and evaluate the range of OTC medicines and therapies used for long COVID self-management. In this study, we surveyed the use of OTC medicines, supplements, remedies, and other therapies to manage symptoms among people with self-reported long COVID in the UK. Our primary objective was to find out the range of treatments individuals with long COVID had used to self-manage symptoms. Our secondary objectives were to explore where these treatments were obtained, factors influencing treatment choices, and associated out-of-pocket expenditure.

## Methods

### Study design

We conducted a cross-sectional electronic survey of adults who had experienced or were experiencing long COVID. The survey was open between 19 January and 5 February 2023 and was accessible to participants residing in the UK. The primary aim was to describe patterns of use of OTC medicines, supplements, remedies and other therapies for the self-management of long COVID symptoms and associated out-of-pocket expenditure.

### Patient and public involvement and engagement

The study was developed in close collaboration with patient partners with lived experience of long COVID. The concept of the research originated from discussions with patient partners who shared their experiences of self-managing their long COVID symptoms, including a vast range of self-management, often costly, practices; inadequate/absent support from healthcare services; and seeking advice from social media platforms. Our partners with lived experience of long COVID were integral to creating the survey content, ensuring its comprehensiveness, testing usability and designing recruitment strategies. Additionally, our patient partners were involved in reviewing and interpreting results and will also be involved in the dissemination of results.

### Participant selection and eligibility

The study invited self-selecting individuals with long COVID, defined as having persistent symptoms such as fatigue for greater than 12 weeks after an episode of acute COVID-19. Individuals were eligible if they were aged 18 years or older with self-reported long COVID and were willing to provide informed consent to participate in the survey.

### Digital platform and data collection

The survey was hosted on REDCap (Research Electronic Data Capture), a secure, web-based software platform for building and managing online surveys and databases, implemented at the University of Birmingham [21, 22]. Data were collected in 2023, and the survey was live from 19/01/2023 to 05/02/2023. Survey items were informed by a review of the literature on long COVID and self-management, clinical experience within the research team, and input from patient partners. The questionnaire included predefined multiple-choice questions and optional free-text responses. Functionality and usability were tested by the research team and patient partners who piloted completion of the survey and provided feedback on ease of completion, visual appearance and the wording of questions and responses. Minor revisions were made before launching the survey.

The survey consisted of three sections (see Supplementary material). Section 1 captures socio-demographic information, including age, gender, ethnicity, geographic location, and current employment status. Section 2 focused on the use of OTC medicines, supplements, remedies, and other therapies, including how much, how often and treatment duration. OTC medicines, supplements and remedies in our survey included analgesics, cough medicines, antacids, decongestants, Steroid nasal sprays, vitamins, antihistamines, sleep medication, and illicit drugs (see Table 1). Alternative therapies in our survey included physiotherapy, acupuncture, aromatherapy, homeopathy, massage therapy, osteopathy, chiropractic, reflexology, hyperbaric oxygen therapy, hypnotherapy, meditation/mindfulness, magnet therapy, yoga, breathwork, counselling, and other therapies. There were also questions about the sources of participants’ OTC medicines, supplements, and remedies, influences on decision making and out-of-pocket expenditure. Section 3 asked about the long-COVID symptoms that participants were attempting to self-manage. Throughout the survey, optional free-text boxes enabled participants to describe additional OTC medicines, therapies or provide further detail.

**Table 1:** List of all OTC medicines, supplements, remedies and other therapies included in the survey

Category	Item
Painkillers	
	Paracetamol
	Ibuprofen
	Co-codamol
	Dihydrocodeine + paracetamol
	Aspirin
	Aspirin
	Any other painkiller medicines
Cough medicine	
	Codeine cough mixture
	Dry cough mixture (e.g. pholcodine, ambroxol)
	Chesty cough mixture (e.g. guaifenesin)
	Any other cough and cold medicines
Antacids/anti-diarrhoeal medicine	
	Alginate (e.g. Gaviscon)
	Antacid (e.g. Rennie)
	Loperamide
	Any other antacids and anti-diarrhoeal medicines
Decongestants	
	Pseudoephedrine
	Decongestant nasal spray
	Steroid nasal spray
	Any other decongestants
Vitamins, minerals and herbal remedies	
	Vitamin D
	Vitamin B
	Vitamin C
	Coenzyme Q10 (CoQ10)
	Zinc
	Curcumin or Turmeric
	Garlic
	Ginger
	Cumin
	Echinacea
	Magnesium
	Multivitamin preparation
	Traditional medicine
	Other vitamin, mineral or herbal supplements
Antihistamines	
	Loratadine
	Cetirizine
	Chlorphenamine
	Acrivastine
	Promethazine
	Other antihistamine
Sleep Medications	
	Melatonin
	Other sleep medicines, please specify
Illicit Substances	
	Cannabis
	Cocaine
	Amphetamine
	CBD oil
	Other illicit drugs

### Participant identification, recruitment and study procedures

Participants were recruited from advertisements (distributed through both newsletters and social media), by word of mouth/known contacts (snowballing), long COVID support groups (including Long Covid SOS [23]), research networks, public/patient involvement websites (e.g. Voice [24]), and the Therapies for Long COVID (TLC) Study website [25]. The advertisements provided a hyperlink to the survey, which first presented a participant information sheet with background information and rationale for the study, what the study participation involved, data protection, and contact details for further information. Participants were required to acknowledge that they had read the participant information sheet before proceeding to an electronic consent form. Only participants who provided consent were given access to the electronic survey.

### Study withdrawals

Participants were able to withdraw at any point during the survey. Before completing the survey, participants were asked to create a unique study number which they could use to request withdrawal of their data from the study for up to one week after completing the survey. Contact details for the study team were provided in the participant information sheet and at the end of the survey.

### Data analysis

#### Quantitative analysis

Quantitative data were analysed using descriptive statistics, including frequencies, proportions, means and standard deviations. The quantitative analysis was performed using Microsoft Excel and Stata SE 15.1. During data cleaning, responses were reviewed for missing, inconsistent, or implausible values, particularly in free-text numeric fields such as expenditure. A small number (8 cost entries from 7 participants) of reported single-session costs were substantially higher than the rest of the cost distribution and were likely to reflect mis-entered totals or package costs rather than individual session costs. To minimise the influence of suspected mis-entries in expenditure data, any reported single-session costs above £200 were capped at £200 before calculating total out-of-pocket expenditure. Missing data were handled using an available-case approach, with the denominator for each analysis corresponding to the number of non-missing observations.

#### Qualitative analysis

Free-text responses from 85 participants, describing additional details on OTC medicine use or therapy experiences, were analysed using CAQDAS (Computer-Aided Qualitative Data Analysis Software), NVivo (QSR International). We conducted an inductive thematic analysis of free-text responses. One researcher (DL) conducted initial open coding of the responses, generating a preliminary coding framework that captured recurring patterns in the data. A second researcher (CM) independently coded 17 out of 85 (20%) responses selected to reflect a range of response types and topics. The two researchers then compared coding, refined the analytic framework through discussion and resolved discrepancies by consensus. The first researcher (DL) subsequently applied the revised framework across the full dataset. Codes were subsequently grouped into higher-order themes that summarised participants’ experiences and rationales for self-management strategies. Themes were developed iteratively by comparing codes and developing themes across participants’ responses to check consistency, refine interpretation and ensure that the final themes reflected the breadth of the data.

## Results

### Quantitative data analysis

#### Participant characteristics

A total of 193 participants consented and answered at least one question in the survey. The demographic characteristics and symptom duration of these participants are reported in Table 2 (and Supplementary Table S1). Most participants were female (153, 79.3%) and identified as White (176, 91.2%). The most common age group was 36–45 years (66, 34.2%), followed by the 46–55 age group (49, 25.4%). Geographically (Supplementary Table S1), the highest proportion of participants were from the Southeast of England (31. 16.1%), Greater London (24, 12.4%) and Scotland (24, 12.4%). Most participants were employed, either full-time (45, 23.3%) or part-time (35, 18.1%). Of those employed full-time or part-time, 45 participants were not working (e.g. due to sick leave). Long COVID symptoms had persisted for more than 12 months in 152 participants (78.8%).

**Table 2:** Participant characteristics (*n* = 193)

	**Number (percentage)**
Gender	
Female	153 (79.3)
Male	36 (18.7)
Non-binary	3 (1.6)
Prefer not to say	1 (0.5)
Age (years)	
18–25	12 (6.2)
26–35	29 (15)
36–45	66 (34.2)
46–55	49 (25.4)
56–65	27 (14.0)
66–75	6 (3.1)
76–85	4 (2.1)
Ethnicity	
White	176 (91.2)
Mixed/Multiple ethnic group	5 (2.6)
Other	5 (2.6)
Asian/Asian British	4 (2.1)
Black/African/Caribbean/Black British	3 (1.6)
Employment	
Employed full-time	45 (23.3)
Employed part-time	35 (18.1)
Employed, but not working	45 (23.3)
Unemployed	25 (13.0)
Voluntary work	1 (0.5)
Full-time education	5 (2.6)
Retired	4 (2.1)
Other	15 (7.8)
Undeclared	18 (9.3)
Duration of symptoms	
3–4 months	4 (2.1)
5–6 months	8 (4.1)
7–8 months	8 (4.1)
9–10 months	9 (4.7)
11–12 months	12 (6.2)
Over 12 months	152 (78.8)

The table showing participants' characteristics about region is displayed in Supplementary Table S1

#### OTC medication use

Table 3 provides an overview of OTC medication use among the 193 participants. Vitamins, minerals and herbal treatments (such as vitamin D, zinc, and traditional medicines) were the most frequently reported OTC products, with 158 of the 178 respondents (88.8%) indicating their use. Analgesics, including paracetamol, ibuprofen and co-codamol, were also commonly reported, with 142 of 193 respondents (73.6%) reporting use. By contrast, substances listed under the illicit substances category (such as cannabis, cocaine, and other illicit drugs) were reported by 10 of 171 respondents (5.8%). Other medications like antacids, antihistamines, and hypnotics (e.g., melatonin) showed moderate usage, suggesting a varied reliance on OTC medicines for health and wellness needs. The "Prefer not to say" responses were infrequent across all categories.

**Table 3:** OTC medication use

	**Responses**	**Number (percentage)**
Analgesics	193	
Yes		142 (73.6)
No		50 (25.9)
Prefer not to say		1 (0.5)
Cough medicine	188	
Yes		24 (12.8)
No		163 (86.7)
Prefer not to say		1.0 (0.5)
Antacids	185	
Yes		63 (34.1)
No		120 (64.9)
Prefer not to say		2 (1.1)
Decongestants	184	
Yes		43 (23.4)
No		140 (76.1)
Prefer not to say		1 (0.5)
Vitamins	178	
Yes		158 (88.8)
No		18 (10.1)
Prefer not to say		2 (1.1)
Antihistamines	172	
Yes		101 (58.7)
No		69 (40.1)
Prefer not to say		2 (1.2)
Hypnotics (sleep medication)	172	
Yes		49 (28.5)
No		122 (70.9)
Prefer not to say		1 (0.6)
Illicit drugs	171	
Yes		10 (5.8)
No		159 (93)
Prefer not to say		2 (1.2)

OTC medicines, supplements and remedies in our survey include analgesics, cough drugs, antacid, decongestant, vitamins, antihistamine, sleep medication, and illicit drugs. More details are listed in Table 1

Supplementary Table S2 shows data on medication overdose, both including and excluding vitamins, minerals and herbal remedies. Overall, 81 participants (42%) reported taking more than the recommended dose in at least one category (including analgesics, cough medications, antacids, decongestants, vitamins, antihistamines, hypnotics and illicit substances). However, this decreased to 39 participants (20.2%) when excluding vitamins, minerals and herbal remedies, indicating that a significant portion of medication overuse was attributable to use of these supplements.

**Fig. 1:**
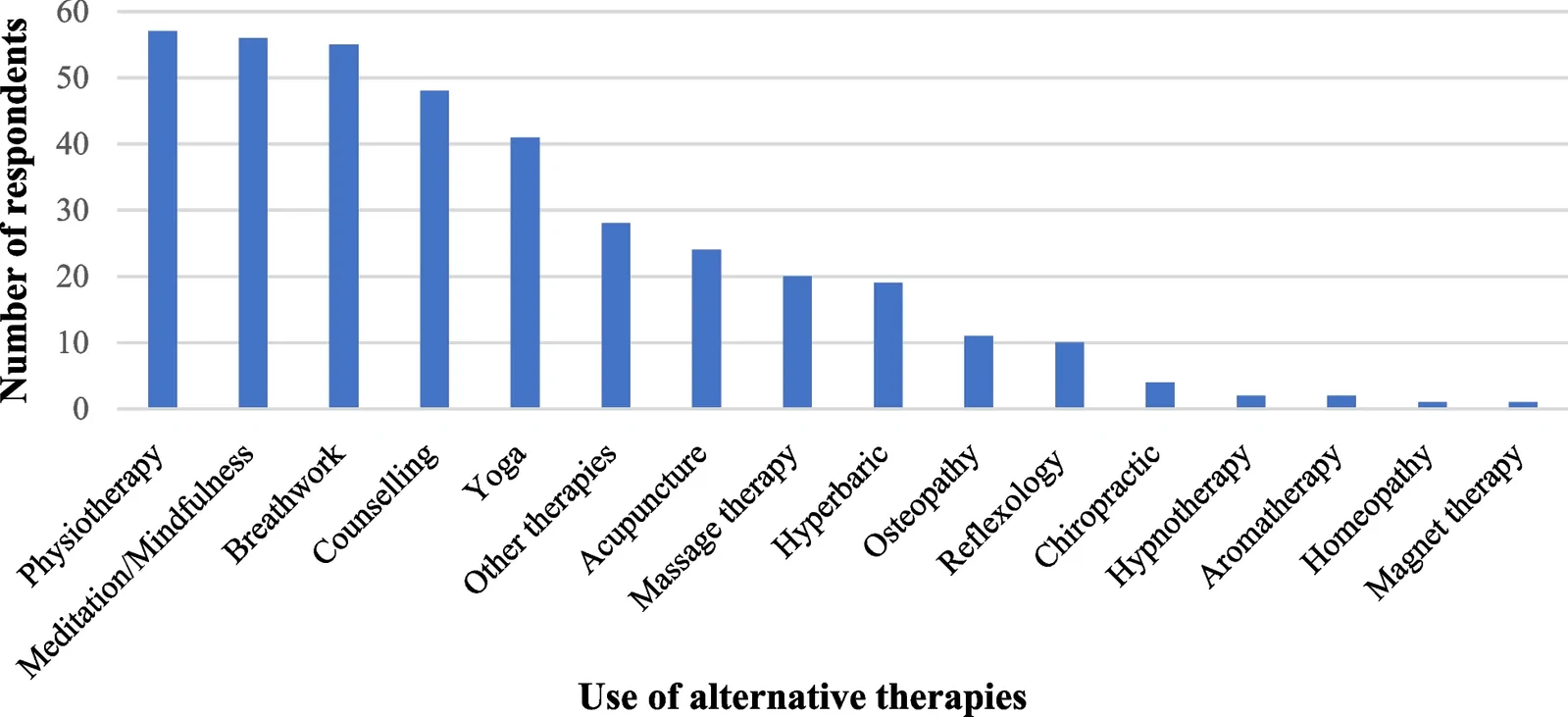
Alternative therapies usage grouped by therapy types. *Notes:* Alternative therapies in our survey include physiotherapy, acupuncture, aromatherapy, homeopathy, massage therapy, osteopathy, chiropractic, reflexology, hyperbaric oxygen therapy, hypnotherapy, meditation/mindfulness, magnet therapy, yoga, breathwork, counselling, and other therapies. The utilisation of different types of alternative therapies is reported in Supplementary Table S3

Use of alternative therapies is summarised in Fig. 1, with full details provided in Supplementary Table S3. Among the 168 participants who completed the corresponding section of the survey, 124 (73.8%) reported using alternative therapies (such as physiotherapy, acupuncture, aromatherapy, homoeopathy, massage therapy, osteopathy, chiropractic, reflexology, hyperbaric oxygen therapy, hypnotherapy, meditation/mindfulness, magnet therapy, yoga, breathwork, counselling, and other therapies). Out-of-pocket expenditure on these therapies (Supplementary Table S4) was reported by 78 of 124 users (62·9%), while 46 (37·1%) reported no personal expenditure. Among those who paid out of pocket, the estimated total expenditure on alternative therapies ranged from £50 to £17 080, with a median of £503 (IQR £201–£1438).

#### Targeted symptoms

**Fig. 2:**
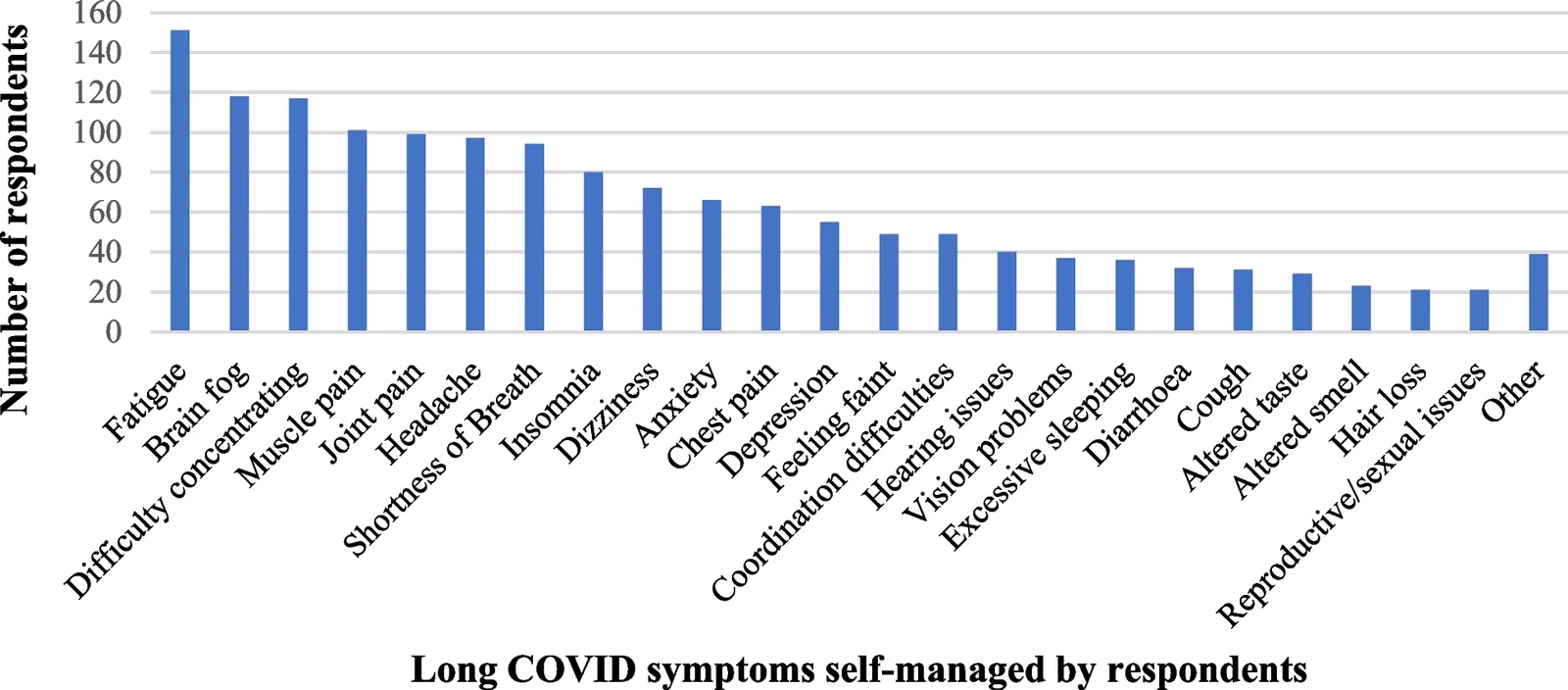
Self-management of long COVID Symptoms. *Notes:* The total number of participants was 165. Participants could report self-managing multiple long COVID symptoms; therefore, symptom categories are not mutually exclusive

Figure 2 shows the range of long COVID symptoms that participants reported managing with OTC medicines, supplements, remedies and/or other therapies. Among the 165 participants, fatigue was the most frequently reported symptom, with 151 participants (91.5%) reporting self-management for it. Muscle pain was reported by 101 participants (61.2%), joint pain by 99 participants (60.0%), and chest pain by 63 participants (38.2%).

#### Factors influencing self-management decisions

Factors influencing decisions about the use of over-the-counter medications, supplements, or remedies to manage long COVID symptoms were reported by 165 participants (Table 4). Online long COVID support groups were the most frequently reported influence, cited by 68 participants (41.2%). Medical doctors were reported as an influencing factor on treatment decision-making by 49 participants (29.7%), followed by social media platforms not specifically associated with long COVID support groups (43, 26.1%), friends or family members (37, 22.4%), and previous experience of treating similar symptoms (24, 14.5%). Other healthcare professionals (e.g., nurses and physiotherapists) were reported by 23 participants (13.9%). However, only 8 participants (4.8%) reported that pharmacists had influenced their treatment decisions. The NHS website was reported as an influencing factor by 11 participants (6.7%), whilst other websites were reported by 21 participants (12.7%). Face-to-face long COVID support groups were reported by 5 participants (3.0%). As participants could select more than one influence, percentages exceed 100%.

**Table 4:** Factors influencing self-management decision making

Factors	Number (percentage)
Online Long COVID support groups	68 (41.2)
Doctors	49 (29.7)
Social media (not including Long COVID support groups)	43 (26.1)
Friends or family members	37 (22.4)
Previous experience of similar symptoms	24 (14.5)
Nurses or allied health professionals (e.g. physiotherapists)	23 (13.9)
Other websites	21 (12.7)
NHS website	11 (6.7)
None	10 (6.1)
Pharmacist	8(4.8)
Face-to-face Long-COVID support groups	5 (3.0)
Other factors	27 (16.4)
Total number of participants	165

As some participants’ decision-making process was influenced by multiple factors, the cumulative percentage exceeds 100%

In total, 43 participants answered the survey question about the social media platforms that had influenced their use of OTC medications, supplements, or home remedies (Supplementary Table S5). X (formerly known as Twitter) was the predominant platform affecting the participants’ self-medication choices, reported by 33 participants (76.7%). Facebook was reported by 16 participants (37.2%). Instagram, TikTok and other platforms were reported less frequently.

Sources of OTC medicines, supplements, and remedies are shown in Supplementary Table S6. Among the 165 respondents, local pharmacies (in person or via delivery) were the most common source (107, 64.8%), followed by high street health shops (83, 50.3%), online pharmacies (59, 35.8%), other online sources (55, 33.3%), and overseas suppliers, whether couriered, purchased during an overseas trip, or bought by family or friends (23, 13.9%).

#### Dietary changes

Participants reported a range of dietary changes adopted to manage their long COVID symptoms (SupplementaryTable S7). Of 169 participants, 110 (61.1%) reported making dietary changes, while 59 (34.9%) reported no changes. Among those who made changes, the most common was becoming teetotal (73, 43.2%), followed by adopting a low-histamine diet such as avoiding avocados (34, 20.1%), engaging in fasting (31, 18.3%), and following a gluten-free diet (25, 14.8%).

### Thematic analysis of free text data

Free-text responses provided additional detail on participants’ experiences of long COVID, the self-management strategies, and the factors influencing their self-management choices. Five themes were identified: the impact of symptoms; other medications used; influences on self-management decisions; dietary changes; and other therapies.

#### The impact of symptoms

Free-text comments were provided by 85 participants in at least one section of the survey. Participants reported a diverse range of over 50 symptoms associated with long COVID in addition to those listed by the survey. The most frequently mentioned were gastro-intestinal symptoms. Other symptoms included neurological symptoms, skin changes, COVID toe (toe swelling and discolouration), and dysphonia. Several participants reported the substantial impact on their working patterns and the burden on their everyday lives, brought on by anxiety, depression, brain fog, and inability to concentrate. They described it as “debilitating”, “hellish”, and “life-wrecking”. They mentioned the unpredictability of the symptoms, their slow improvement, and the need for coping strategies. The fluctuating nature of symptoms and the challenges of reinfection were also highlighted by participants.

*“The depression and anxiety are the result of dealing with this illness, existing rather than living” (P40)*

*“After 2 years I have managed to read a book cover to cover which has perked me up enormously. Brain fog and the inability to concentrate add that to disrupted sleep and then sleeping in afternoons has been awful.” (P34)*

#### Other medication used

Participants commented on the use of a wide variety of other medications to manage long COVID symptoms. For example, participants reported using various forms of analgesia, such as nonsteroidal anti-inflammatory drugs (NSAIDs), neuropathic pain relief medication, opiates, and cannabidiol (CBD). In addition, some reported using combination drugs (i.e. drugs that contain more than one active ingredient, such as co-codamol). There were mentions of the use of cough medication including throat sprays and throat sweets, and decongestants. The use of proton pump inhibitors (antacids) was also frequently mentioned. Participants reported using various vitamins, minerals, herbal or traditional remedies, probiotics, and a wide range of supplements. Other medications such as amitriptyline, propranolol, and tranexamic acid were also mentioned by participants. Finally, antihistamines were mentioned by several participants, either for standard use (i.e. to manage allergy symptoms) or as a hypnotic.

Some participants reported taking medication to self-manage long COVID symptoms that had been prescribed for another condition. Some participants had previously or were currently trialling medication and self-assessing its impact on their symptoms. Comments on the effectiveness of medications used ranged from no effect at all, to dramatic symptom improvements. Participants also reported obtaining OTC medication through a variety of online stores.

*“The naproxen I took was prescribed by my GP for back pain which came on at time of primary COVID illness. I already took multivitamins, probiotics and cod liver oil supplements before covid so continued to take these. I also periodically used antihistamines for allergies and hay fever anyway.” (P168)*

*“Fexofenadine helps my neuro symptoms.” (P3)*

*“I also take the YourGut+ probiotic. The codeine often doesn't work.” (P7)*

#### Influences on self-management decisions

Participants were asked about the sources influencing their decision to use non-prescribed medicines for managing long COVID symptoms. Regarding information sources, health professionals' advice and information from research papers were commonly mentioned. Other sources included health information websites, books, podcasts, newspapers, or advice from peers or therapists. Long COVID support groups were also frequently mentioned, particularly those based online.

Some participants highlighted a sense of despair due to persistent symptoms and the lack of reliable information or effective treatments, when they make the decision to use non-prescribed medication. Many felt unsupported by the healthcare system, with feelings of being ignored or dismissed, leading to self-directed research and trials of different medications. Some expressed a willingness to try anything to improve symptoms, driven by a lack of prescribed options and frustration with mainstream healthcare routes. Some reported trying non-prescribed medications based on their personal experience without any professional support, given the lack of official information, limited access to healthcare services, and lack of definitive treatments from GPs; while others received advice or support from peers or healthcare professionals.

*“I feel desperate.” (P22)*

*“I am desperate to get better, and so doing something simple like taking vitamin D is worth a try.” (P94)*

*“There is a dearth of information and it’s scary.” (P13)*

*“My life is unrecognisable from the one I lived before; I can't walk up a few steps without getting breathless and my fatigue is often insurmountable, and my brain fog was so bad I thought I might be getting dementia, I would try anything” (P205)*

*“I was very skeptical about taking recommendations from randoms on the internet but as I had had nothing useful suggested through mainline NHS routes… just tried it out of desperation! ....” (P11)*

#### Dietary changes

Participants mentioned a wide range of other dietary changes to improve their long COVID symptoms, including reducing consumption of sugar, meat (including going vegan) and carbohydrates, and going on a keto diet. Others mentioned low FODMAP (fermentable oligosaccharides, disaccharides, monosaccharides, and polyols) diets and reduced meal sizes.

Several participants reported that dietary changes had improved their symptoms, ranging from improvements in abdominal pain and bloating to breathlessness and energy levels. Others noted how certain substances (predominantly alcohol) worsened their symptoms. Difficulty in maintaining dietary changes was also reported by some participants, particularly concerning a low histamine diet. Participants felt there was a lack of information from the health service about the role of diet in long COVID and their dietary changes were based on personal research and recommendations from other long COVID sufferers among long COVID support groups, particularly via social media.

*"Diet changes have relieved me of many symptoms and made life bearable again. NHS advise not to change diet. I'm glad I didn't listen." (P119)*

When commenting on what had influenced them to make changes to their diet, one respondent answered:

*“The desire to get well enough to work... It is very hard trying to work out what to take and to know how to experiment safely. I wish there were some guidance." (P84)*

#### Other therapies

Participants mentioned twenty other therapies used to manage long COVID symptoms, including cognitive behavioural therapies, relaxation therapies, and cryotherapies. Decisions to access these therapies were influenced by online support groups, previous experience, workplace recommendations and previous positive experiences. A lack of alternative support from the healthcare system was a common reason cited for seeking these therapies, with issues ranging from the perceived unwillingness of healthcare professionals to acknowledge people's needs and offer management plans, to limited treatment options and long waiting times. The need for support and care plans was emphasised by participants.

*“Again, all motivated because of a lack of support, care plan or willingness to research treatment by my GP” (P7) *

*“Ended up consulting with functional medicine doctor through a mutual friend and because I felt I wasn't getting anywhere with NHS services” (P125)*

*“No other choice as huge waiting lists so have to try and live with the illness” (P136)*

In terms of the impact of these other therapies, participants expressed mixed feelings: some found partial relief, while others experienced limited or no impact of these alternative therapies.

“*I've tried DIY meditation at home but it doesn't cut it—might make me feel a bit better mentally but doesn't get rid of the underlying physical symptoms….” (P13).*

*“Meditation with the Sensate device works best to improve symptoms. ENO breathing course helped too.” (P16)*

*“Yoga, meditation and breath work are helping me manage the roller coaster that is this illness. acupuncture may or may not be doing anything. Curable, I have just started, as I figured anything was worth a try at this point.” (P139).*

## Discussion

In this study, we surveyed the use of self-directed OTC medicines, dietary supplements, herbal remedies, and non-pharmacological therapies based on a group of UK-based individuals with self-reported long COVID symptoms. The most common long COVID symptoms that participants self-managed were fatigue, brain fog, difficulty concentrating, chest pain, joint pain, headache, shortness of breath, and gastrointestinal issues. The most frequently used types of OTC medicines and supplements were vitamins, analgesics, and antihistamines, with a notable proportion of participants exceeding recommended dosages. The most preferred sources of acquiring self-medicated drugs were pharmacies (either in person or online) and high street shops. A substantial number of participants had also tried alternative non-pharmacological therapies (e.g., physiotherapy, meditation, breathwork, and yoga) and made dietary changes to manage long COVID symptoms. Among those who incurred out-of-pocket expenditure for alternative therapies, the total expenditure ranged from £50 to £17,080, with a median expenditure of £503. Friends, family members, medical professionals, online Long COVID support groups, and social media platforms were perceived to play important roles in decision-making for self-care and self-medication.

The qualitative findings provided additional context for these patterns. Participants reported additional symptoms beyond those captured in the structured survey, with gastrointestinal complaints most frequently mentioned alongside neurological symptoms and dermatological changes. Participants described long COVID as disruptive, unpredictable and burdensome, affecting daily activities, work, mood, sleep and concentration. Free-text responses also suggested that self-management was often shaped by persistent and fluctuating symptoms, uncertainty about recovery, perceived gaps in healthcare support, limited access to effective treatment options, personal research, and peer advice. Perceived effectiveness of self-management approaches varied across participants, ranging from no benefit to substantial symptom improvement. These findings help explain why participants used multiple self-management strategies and the wider impact of long COVID on daily life.

According to the findings of a systematic review by O’Mahoney et al. (2022), the most prevalent symptoms in non-hospitalised patients with long COVID were fatigue, breathlessness, muscle pain, insomnia and loss of smell [3]. We found that the most self-managed long COVID symptoms among our participants were fatigue, brain fog, difficulty concentrating, chest pain, joint pain, headache, shortness of breath, and gastro-intestinal issues. The overlap and differences highlight the multifaceted long-term impact of COVID-19 on individuals’ experience of symptoms and may reflect the self-selected nature of survey participants, many of whom had long-term symptoms. Symptoms such as fatigue, brain fog, difficulty concentrating, and muscle and joint pain, can severely disrupt daily activities, work, and social interactions. These symptoms impose a continuous burden on patients, leading to significant challenges in managing daily life, and can negatively impact quality of life [26]. However, with limited access to long COVID care from healthcare services, these symptoms have been commonly self-managed using OTC medicines and other therapies [27].

We found that the most frequently used OTC medicines and dietary supplements were vitamins, analgesics, and antihistamines, which is consistent with emerging evidence on long COVID self-management [28]. Koss and Bohnet-Joschko used social media data to explore medications and supplements reported by people self-treating long COVID symptoms and identified frequent discussion of histamine antagonists, magnesium, vitamins, and steroids. Evidence from the wider COVID-19 literature also shows that self-medication was common during the COVID-19 pandemic, with analgesics, antibiotics, and nutritional supplements frequently reported for prevention or treatment of acute COVID-19 symptoms [29–32]. However, most existing studies have focused on self-medication for acute infection or prevention, and their findings may not be directly applicable to long COVID because of differences in symptom profile, duration, and management needs. Our findings therefore extend existing evidence by characterising the range of non-prescribed medicines and supplements used specifically for long COVID self-management in a UK sample.

Many of our study participants reported acquiring information on self-medication from friends, family members, medical professionals, online long COVID support groups, social media platforms and websites (e.g., scientific journal-related websites, science reports, etc.), which played important roles in decision-making for self-care and self-medication. Self-medication practices were common across the world during the COVID-19 pandemic. Although not specifically focusing on long COVID management, previous studies [33, 34] have also underlined the critical role of the internet (e.g., online communities, social media, online peer support), suggestions from friends or family members [35], previous experiences of treating similar symptoms, and advice from friends or family members who are healthcare professionals [36], in shaping health decision making and behaviours among people with various health conditions. Amongst those factors influencing decision-making about OTC medications, the Internet has increasingly become the predominant source of health information during the COVID pandemic. This was partly due to fears of nosocomial infection, restricted access to healthcare services (e.g., experiencing long waiting times for care) and patients feeling unsupported [34, 37, 38].

With the current lack of effective treatments available for long COVID, there seems to have been a similar trend toward self-prescription and other forms of self-management based on information from various online sources. However, people with long COVID who actively seek health information from these sources risk being exposed to outdated and misinterpreted information [33]. Without adequate health literacy and the guidance of healthcare professionals, the use of the internet to inform self-medication carries risks of harm from potential medication misuse and adverse health effects [33].

We observed that individuals with long COVID self-medicated with vitamin supplements, analgesics, antihistamines, and other non-prescribed products, even though it is unclear whether those treatments are effective and safe in managing long COVID symptoms, given the lack of confirmed clinical evidence [39]. Participants reported varied perceived effects, ranging from no benefit to substantial symptom improvement, and some also mentioned adverse effects. This aligns with the mixed and product-specific nature of the emerging evidence. Some non-prescribed products, such as L-arginine plus vitamin C and the synbiotic preparation (SIM01), have shown potential benefit; whereas others (e.g., high-dose coenzyme Q10 and lactoferrin) have shown no clear benefit for long COVID symptom relief [40–43]. Nevertheless, we still lack robust clinical evidence on the effectiveness and safety of most non-prescribed products used by individuals with long COVID, suggesting directions for future research.

Some of the self-management approaches reported by participants, particularly physiotherapy, breathing exercises, pacing and other rehabilitation- or exercise-based non-pharmacological therapies, overlap with approaches for which there is an emerging evidence base in long COVID. Recent evidence syntheses suggest that physical and mental health rehabilitation may improve some long COVID symptoms, and that exercise-based or pulmonary rehabilitation may improve fatigue, breathlessness, physical capacity, and health-related quality of life in some patients, although the evidence remains heterogeneous [44–46]. Evidence for rehabilitation targeted at cognitive symptoms such as “brain fog” and difficulty concentrating is promising but still preliminary [47]. These findings underline the need for further evidence on the effectiveness of non-pharmacological therapies and for evidence-informed guidance to support people who use those approaches to manage long COVID symptoms.

The risks associated with inappropriate use of OTC medication (e.g., from overdosing) should be highlighted. For instance, paracetamol overdoses can result in liver damage [48]. Excess vitamin D intake can lead to certain symptoms of hypercalcemia including fatigue, weakness, anorexia, nausea, vomiting, and polyuria [49]. Our finding that a notable proportion of participants exceeded recommended dosages of OTC medications and supplements indicates that health information and guidance should be provided to people with long COVID to support decisions on self-medication. Furthermore, significantly more evidence is needed to determine the effect of these medicines, supplements, and other therapies for the management of long COVID symptoms.

Our study has several strengths. It assessed a comprehensive range of self-management behaviours for long COVID symptoms, including the use of OTC medicines, dietary supplements, herbal remedies, and non-pharmacological therapies. It is one of the few studies that has explored this topic in detail among adults with long COVID. Our study provides a foundation for further research on self-management behaviours among people with long COVID, which can be used by policymakers, researchers, and healthcare professionals to inform publicly available information about long COVID management, clinical guidelines, and training for healthcare professionals.

Another strength is that our survey assessed self-medication using both a pre-defined list of medicines, as well as open questions where participants could provide further information, ensuring that we captured a wide breadth of self-treatment practices. Third, we captured and analysed both quantitative and qualitative data. The descriptive quantitative analysis provided an overview of the prevalence of self-medication and the use of other non-prescribed therapies among our survey respondents. Complementary to this, the thematic analysis of free-text responses provided a more in-depth understanding and description of personal experiences and behaviours regarding the self-management of long COVID, as well as the reasoning behind why some people resorted to these alternative therapies. Including both quantitative and qualitative information offered deeper insights than either approach alone.

However, data in our study were collected by an online survey, which poses a risk of selection bias, including the exclusion of people without reliable access to the internet. Most participants were female, mainly employed and predominantly identified as belonging to a white ethnic background. While this limits representativeness, it is consistent with published evidence showing that women are disproportionally affected by long COVID and more likely to report persistent symptoms than men [50]. Nevertheless, the gender and ethnic distribution in our sample may still constrain the generalisability of the findings to the wider UK population affected by long COVID. Secondly, our study included a relatively small sample size, again limiting generalisability. Thirdly, the study was not designed to include an integrated synthesis of quantitative and qualitative data, which is in line with the original research plan. A more explicitly integrated mixed-methods approach could be explored in future research. Finally, our survey did not systematically seek to capture data on whether medications were perceived to have been effective or to capture data on adverse effects, which are areas for future research.

Our findings underscore the need for effective, regulated treatments to manage long COVID symptoms in the UK. In the absence of such treatments, people with long COVID will understandably continue to seek alternative treatments, which include OTC medicines, dietary supplements, herbal remedies, and alternative therapies. It is important to consider the global implications of these findings, particularly in regions where OTC medication and other therapies are less regulated. Information about these non-prescribed therapies should be provided on trusted online websites that have been vetted and quality-assured by healthcare providers and policymakers. Such sources should include easily accessible information on potential benefits, harms, and recommendations on safe and appropriate use of treatments. Healthcare professionals supporting people with long COVID should be aware of the wide landscape of non-prescribed therapies that their patients may be accessing, ascertain information in their history about self-management practices, and offer balanced information and guidance on their safe and appropriate use. This should also be accounted for when prescribing medicines to avoid potential adverse drug interactions and to monitor for harm from non-prescribed treatments. High street pharmacists may play an important role in this as people with long COVID are likely to source non-prescribed treatments from commercial pharmacies.

Future studies should consider the findings of this survey when investigating therapies for long COVID, including pre-defined data capture on non-prescribed treatments that research participants may be accessing. There is also a need to examine self-management practices in a larger and more diverse and representative cohort of people with long COVID, and to evaluate both the effectiveness, harms, and costs of the main OTC medicines, dietary supplements, and non-pharmacological interventions identified in this survey. Investigating the psychological aspects of long COVID and its management, especially the role of online support groups and peer influence on self-management practices, would further contribute to the field.

## Conclusion

People with long COVID may access a wide range of OTC medicines, dietary supplements, herbal remedies, and non-pharmacological therapies to self-manage symptoms. This includes vitamin supplements, analgesics, and antihistamines, sometimes exceeding recommended doses, and are often acquired from online and in-person pharmacies and high street shops. Other common self-management practices include using alternative non-pharmacological therapies (e.g., physiotherapy) and dietary changes. A wide range of factors influence self-management choices such as friends, family members, medical professionals, online support groups, and social media platforms. Healthcare providers should be aware of the use of non-prescribed therapies among long COVID sufferers and consider these in their treatment plans. Public health policies should focus on providing accurate information and guidance for patients self-managing long COVID symptoms.

## Supplementary Information

Supplementary Material 1.

## Data Availability

The collected data used in the current study are available from the corresponding author upon reasonable request.
