# Supplementary material for: Self-management of long COVID symptoms with over-the-counter medicines and other non-prescribed therapies: a cross-sectional survey
Source: BMC Public Health. 2026 Jul 2;26:2643. doi: 10.1186/s12889-026-28017-5 (PMC13595737; doi:10.1186/s12889-026-28017-5)
Supplement: Supplementary file 1 [file 12889_2026_28017_MOESM1_ESM.docx]

**Supplementary Material**

**Self-management of long COVID symptoms with over-the-counter medicines and other non-prescribed therapies: a cross-sectional survey**

Authors: Naijie Guan, Grace Turner, Richard Hotham, Daniel Lange, Kirsty R Brown, Christel McMullan, Sarah E Hughes, Olalekan Lee Aiyegbusi, Karen Matthews, Louise Jackson, Asma Yahyouche, Yvonne Alder, Felicity Jeyes, Lewis Buckland, Amy Chong, David Stanton, Melanie Calvert & Shamil Haroon

**Correspondence:**

Naijie Guan: [n.guan@bham.ac.uk](mailto:n.guan@bham.ac.uk) & Shamil Haroon: [s.haroon@bham.ac.uk](mailto:s.haroon@bham.ac.uk)

# **Additional file 1 Results Tables**

### **Table S1 Participant characteristics: Region (n=193)**

|  | **Number (percentage)** |
| --- | --- |
| **Region** |  |
| North East | 3 (1.6) |
| North West | 17 (8.8) |
| Yorkshire and the Humber | 17 (8.8) |
| West Midlands | 19 (9.8) |
| East Midlands | 13 (6.7) |
| South West | 17 (8.8) |
| South East | 31 (16.1) |
| East of England | 10 (5.2) |
| Greater London | 24 (12.4) |
| Scotland | 24 (12.4) |
| Northern Ireland | 9 (4.7) |
| Wales | 7 (3.6) |
| Prefer not to say | 2 (1.0) |

### **Table S2 Medication overuse**

| **Medication overuse** (Did the person report taking above the recommended dose) | Number (percentage) |
| --- | --- |
| Yes | 81 (42.0) |
| No | 112 (58.0) |
| Total number of respondents | 193 |
| **Medication overuse excluding vitamins** (Did the person report taking above the recommended dose) |  |
| Yes | 39 (20.2) |
| No | 154 (79.8) |
| Total number of respondents | 193 |

### **Table S3 Alternative therapies used**

| **Alternative therapies used** | **Number (percentage)** |
| --- | --- |
| Physiotherapy | 57 (33.9) |
| Meditation/Mindfulness | 56 (33.3) |
| Breathwork | 55 (32.7) |
| Counselling | 48 (28.6) |
| Yoga | 41 (24.4) |
| Other therapies | 28 (16.7) |
| Acupuncture | 24 (14.3) |
| Massage therapy | 20 (11.9) |
| Hyperbaric oxygen therapy | 19 (11.3) |
| Osteopathy | 11 (6.6) |
| Reflexology | 10 (6.0) |
| Chiropractic | 4 (2.4) |
| Hypnotherapy | 2 (1.2) |
| Aromatherapy | 2 (1.2) |
| Homeopathy | 1 (0.6) |
| Magnet therapy | 1 (0.6) |
| Total number of respondents | 168 |

*Notes:* Some participants used multiple types of alternative therapies.

### **Table S4 Costs of alternative therapies**

| **Alternative therapies used** | **Number (percentage) of participants reporting out-of-expenditure expenditure** | **Range of out-of-pocket expenditure per session (£)** |
| --- | --- | --- |
| Physiotherapy | 11 (8.9) | 20-55 |
| Acupuncture | 22 (17.7) | 25-85 |
| Aromatherapy | 1 (0.8) | 45-45 |
| Homeopathy | 1 (0.8) | 70-70 |
| Massage therapy | 19 (15.3) | 35-100 |
| Osteopathy | 9 (7.3) | 45-150 |
| Chiropractic | 4 (3.2) | 40-120 |
| Reflexology | 10 (8.1) | 20-50 |
| Hyperbaric oxygen therapy | 18 (14.5) | 10-200 |
| Hypnotherapy | 1 (0.8) | 75-75 |
| Meditation/mindfulness | 8 (6.5) | 0-75 |
| Magnet therapy | 1 (0.8) | 70-70 |
| Yoga | 20 (16.1) | 5-100 |
| Breathwork | 9 (7.3) | 0-200 |
| Counselling | 16 (12.9) | 10-200 |
| Other | 15 (12.1) | 12-200 |
| No costs | 48 (38.7) | NA |
| Prefer not to say | 2 (1.6) | NA |

*Notes:* Total number of participants reported accessing alternative therapies for Long COVID management: 124. Total number of participants reported spending their own money on those alternative therapies: 78. Cost ranges refer to reported single-session, single-course or single-subscription costs. Values above £200 were capped at £200 before calculating total out-of-pocket expenditure.

### **Table S5 Social media platforms reported to have influenced self-medication decisions**

| **Social media platforms** | **Number (percentage)** |
| --- | --- |
| Twitter | 33 (76.8) |
| Facebook | 16 (37.2) |
| TikTok | 1 (2.3) |
| Instagram | 5 (11.6) |
| Other | 5 (11.6) |
| Total number of respondents | 43 |

*Notes:* As some respondents use multiple social media platforms, the cumulative percentage exceeds 100%.

### **Table S6 Source of medicines, supplements, and remedies**

| **Sources** | **Number (percentage)** |
| --- | --- |
| Pharmacy - local (in person/delivery) | 107 (64.8) |
| Pharmacy - online | 59 (35.8) |
| High street shop | 83 (50.3) |
| Another country e.g. couriered, brought back from trip or brought back by family/ friend | 23 (13.9) |
| Online other | 55 (33.3) |
| Total number of respondents | 165 |

*Notes:* As some respondents use multiple sources, the cumulative percentage exceeds 100%.

### **Table S7 Dietary changes to manage long COVID symptoms**

| **Dietary Changes** | **Number (percentage)** |
| --- | --- |
| Gluten-Free | 25 (14.8) |
| Food low in histamine (e.g. avocados) | 34 (20.1) |
| Alcohol-free | 73 (43.2) |
| Dairy Free | 25 (14.8) |
| Fasting | 31 (18.3) |
| Other | 39 (23.1) |
| No changes | 59 (34.9) |
| Total number of respondents | 169 |

*Notes:* As some respondents had more than one type of dietary change, the cumulative percentage exceeds 100%.

# **Additional file 2 Survey Questionnaire**

**Survey of non-prescribed medicines, supplements, remedies and other therapies for Long COVID**

*Memorable ID number*

Please create a memorable ID number made up of:

- The last 3 digits of your telephone number (e.g. 707)
- The numerical parts of your postcode (e.g. 461)

Your final ID number should have 5 or 6 numbers (e.g. 707461)

_____________________

Please write down this number for your records.

### Section 1: Information about you

1. **What is your age?**
   a. 18-25 years
   b. 26-35 years
   c. 36-45 years
   d. 46-55 years
   e. 56-65 years
   f. 66-75 years
   g. 76-85 years
   h. 86-95 years
   i. ≥95 years
   j. Prefer not to say
2. **Are you?**
   a. Male
   b. Female
   c. Non-binary
   d. Prefer not to say
3. **What is your ethnicity?**
   a. White
   b. Asian/Asian British
   c. Black/African/Caribbean/Black British
   d. Mixed/Multiple ethnic group
   e. Other, please specify
   f. Prefer not to say
4. **Where do you live?**
   a. North East
   b. North West
   c. Yorkshire and the Humber
   d. West Midlands
   e. East Midlands
   f. South West
   g. South East
   h. East of England
   i. Greater London
   j. Scotland
   k. Northern Ireland
   l. Wales
   m. Prefer not to say
5. **What is your current employment status? (Select all that apply)**
   a. Employed – full time
   b. Employed – part time
   c. Employed but currently not working; for example sick leave
   d. Unemployed
   e. Furloughed
   f. Voluntary work
   g. In full time education
   h. Retired
   i. Prefer not to say
   j. Other, please specify: __________________
6. **How long have you had COVID symptoms?**
a. 3-4 months
b. 5-6 months
c. 7-8 months
d. 9-10 months
e. 11-12 months
k. Over 12 months

### Section 2: Managing Long COVID symptoms

The following questions will ask about use of non-prescribed over-the-counter medications and other therapies to manage Long COVID symptoms. Please note, we do NOT endorse the use of any of the medicines or therapies listed for the management of Long COVID.

**Don’t worry if you can’t remember all the medicines, supplements, remedies and other therapies you have tried, any information is helpful.**

Non-prescribed medicines, supplements and remedies

**Have you used any of the following non-prescribed medicines, supplements or remedies to help manage your Long COVID symptoms?**

**A) Painkillers**

|  | Yes (tick box) | How much did you take each time? (i.e. how many tablets, sprays, applications) | How many times a week did you take this medication? | How long have you used this medication for? (days/ weeks/ months) | Have you taken this in the last week? |
| --- | --- | --- | --- | --- | --- |
| Paracetamol |  |  |  |  |  |
| Ibuprofen (200mg) |  |  |  |  |  |
| Ibuprofen (400mg) |  |  |  |  |  |
| Co-codamol |  |  |  |  |  |
| Paramol (dihydrocodeine + Paracetamol) |  |  |  |  |  |
| Aspirin (75mg) |  |  |  |  |  |
| Aspirin (300mg) |  |  |  |  |  |
| Any other painkiller medicine, please specify |  |  |  |  |  |

**B) Cough medicines**

|  | Yes (tick box) | How much did you take each time? (i.e. how many tablets, sprays, applications) | How many times a week did you take this medication? | How long have you used this medication for? (days/ weeks/ months) | Have you taken this in the last week? |
| --- | --- | --- | --- | --- | --- |
| Codeine Cough Mixture |  |  |  |  |  |
| Dry Cough Mixture (e.g. Pholcodine, Ambroxol) |  |  |  |  |  |
| Chesty Cough Mixture (e.g. Guaifenesin) |  |  |  |  |  |
| Any other Cough and cold medicine, please specify |  |  |  |  |  |

**C) Antacids and anti-diarrhoea medicines**

|  | Yes (tick box) | How much did you take each time? (i.e. how many tablets, sprays, applications) | How many times a week did you take this medication? | How long have you used this medication for? (days/ weeks/ months) | Have you taken this in the last week? |
| --- | --- | --- | --- | --- | --- |
| Gaviscon (tablets, liquid) |  |  |  |  |  |
| Loperamide (Imodium) |  |  |  |  |  |
| Rennie |  |  |  |  |  |
| Any other antacids and anti-diarrhoea medicines, please specify |  |  |  |  |  |

**D) Decongestants**

|  | Yes (tick box) | How much did you take each time? (i.e. how many tablets, sprays, applications) | How many times a week did you take this medication? | How long have you used this medication for? (days/ weeks/ months) | Have you taken this in the last week? |
| --- | --- | --- | --- | --- | --- |
| Pseudoephedrine |  |  |  |  |  |
| Decongestant Nasal Spray (e.g. Otrivine) |  |  |  |  |  |
| Steroid nasal spray (e.g. Beconase, Pirinase) |  |  |  |  |  |
| Any other decongestant, please specify |  |  |  |  |  |

**E) Vitamins, minerals and herbal remedies**

|  | Yes (tick box) | How much did you take each time? (i.e. how many tablets, sprays, applications) | How many times a week did you take this medication? | How long have you used this medication for? (days/ weeks/ months) | Have you taken this in the last week? |
| --- | --- | --- | --- | --- | --- |
| Vitamin D |  |  |  |  |  |
| Vitamin B |  |  |  |  |  |
| Vitamin C |  |  |  |  |  |
| Coenzyme Q10 (CoQ10) |  |  |  |  |  |
| Zinc |  |  |  |  |  |
| Curcumin or Turmeric |  |  |  |  |  |
| Garlic |  |  |  |  |  |
| Ginger |  |  |  |  |  |
| Cumin |  |  |  |  |  |
| Echinacea |  |  |  |  |  |
| Magnesium |  |  |  |  |  |
| Multivitamin preparation |  |  |  |  |  |
| Traditional medicine, please specify |  |  |  |  |  |
| Other vitamin, mineral or herbal supplements, please specify |  |  |  |  |  |

**F) Antihistamines**

|  | Yes (tick box) | How much did you take each time? (i.e. how many tablets, sprays, applications) | How many times a week did you take this medication? | How long have you used this medication for? (days/ weeks/ months) | Have you taken this in the last week? |
| --- | --- | --- | --- | --- | --- |
| Loratadine (Claritin) |  |  |  |  |  |
| Cetirizine |  |  |  |  |  |
| Chlorphenamine (Piriton) |  |  |  |  |  |
| Acrivastatine (Benadryl) |  |  |  |  |  |
| Promethazine (Phenergan) |  |  |  |  |  |
| Other antihistamine, please specify |  |  |  |  |  |

**G) Sleep medicines**

|  | Yes (tick box) | How much did you take each time? (i.e. how many tablets, sprays, applications) | How many times a week did you take this medication? | How long have you used this medication for? (days/ weeks/ months) | Have you taken this in the last week? |
| --- | --- | --- | --- | --- | --- |
| Melatonin |  |  |  |  |  |
| Other sleep medicines, please specify |  |  |  |  |  |

**H) Illicit drug**

|  | Yes (tick box) | How much did you take each time? (i.e. how many tablets, sprays, applications) | How many times a week did you take this medication? | How long have you used this medication for? (days/ weeks/ months) | Have you taken this in the last week? |
| --- | --- | --- | --- | --- | --- |
| Cannabis |  |  |  |  |  |
| Cocaine |  |  |  |  |  |
| Amphetamine |  |  |  |  |  |
| CBD oil |  |  |  |  |  |
| Other illicit drug, please specify |  |  |  |  |  |

**I) Other**

|  | Yes (tick box) | How much did you take each time? (i.e. how many tablets, sprays, applications) | How many times a week did you take this medication? | How long have you used this medication for? (days/ weeks/ months) | Have you taken this in the last week? |
| --- | --- | --- | --- | --- | --- |
| Other, please specify |  |  |  |  |  |

Any comments?

**Where did you get your medicines, supplements or remedies from? (tick all that apply)**

a) Pharmacy – in person
b) Pharmacy – online
c) High street shop, e.g. Holland & Barrett
d) Another country e.g. couriered, brought back from trip or brought back by family/ friend
e) Online, please specify

**What influenced your decision to use non-prescribed medicines, supplements or remedies to manage your Long COVID symptoms?**

a) Friends or family members
b) Pharmacist
c) Doctor
d) Nurse or allied health professional (e.g. physio)
e) NHS website
f) Other websites, please specify
g) Online Long COVID support group, please specify
h) Face-to-face Long-COVID support group, please specify
i) Social media (***not*** Long COVID support group)
   a. Twitter
   b. Facebook
   c. Tiktok
   d. Instagram
   e. Other, please specify
j) Previous experience of similar symptoms
k) Other, please specify

Any comments?

**Changes to your diet**

**Have you made any of the following changes to your diet to help manage long COVID symptoms?**

- Gluten free
- Foods low in histamines e.g. avocadoes
- Alcohol free
- Dairy free
- Fasting
- Other, please specify

Any comments?

**What influenced your decision to change your diet to manage your Long COVID symptoms?**

a) Friends or family members
b) Pharmacist
c) Doctor
d) Nurse or allied health professional (e.g. physio)
e) NHS website
f) Other websites, please specify
g) Online Long COVID support group, please specify
h) Face-to-face Long-COVID support, please specify
i) Social media (***not*** Long COVID support group)
   a. Twitter group
   b. Facebook
   c. Tiktok
   d. Instagram
   e. Other, please specify
j) Previous experience of similar symptoms
k) Other, please specify

Any comments?

**Other therapies**

**Have you used any of the following therapies to help manage your Long COVID symptoms?**

|  | **Yes** | **NHS/paid for/free** | **How many sessions have you had?** |
| --- | --- | --- | --- |
| Physiotherapy |  |  |  |
| Acupuncture |  |  |  |
| Aromatherapy |  |  |  |
| Homeopathy |  |  |  |
| Massage therapy |  |  |  |
| Osteopathy |  |  |  |
| Chiropractic |  |  |  |
| Reflexology |  |  |  |
| Hyperbaric oxygen therapy |  |  |  |
| Hypnotherapy |  |  |  |
| Meditation/mindfulness |  |  |  |
| Magnet therapy |  |  |  |
| Yoga |  |  |  |
| Breathwork |  |  |  |
| Counselling |  |  |  |
| Other, please specify |  |  |  |
| None |  |  |  |

Any comments

**What influenced your decision to use these therapies to manage your Long COVID symptoms?**

a) Friends or family members
b) Pharmacist
c) Doctor
d) Nurse or allied health professional (e.g. physio)
e) NHS website
f) Other websites, please specify
g) Online Long COVID support group, please specify
h) Face-to-face Long-COVID support group, please specify
i) Social media (***not*** Long COVID support group)
   a. Twitter
   b. Facebook
   c. TikTok
   d. Instagram
   e. Other, please specify
j) Previous experience of similar symptoms
k) Other, please specify

Any comments

### Section 3: Long COVID symptoms

**Which Long COVID symptoms were you trying to manage with non-prescribed medicines, supplements, remedies or other therapies?**

a) Fatigue (tiredness)
b) Chest pain
c) Joint pain
d) Muscle pain
e) Headache
f) Cough
g) Shortness of breath
h) Diarrhoea
i) Altered taste
j) Altered smell
k) Depression
l) Anxiety
m) Feeling faint
n) Dizziness
o) Brain fog
p) Difficulty concentrating
q) Coordination difficulties
r) Insomnia - lack of sleep
s) Excessive sleeping
t) Ear/hearing issues
u) Hair loss
v) Eye/vision issues
w) Reproductive or sexual health issues
x) Other, please specify

Any comments?

### Section 4: Comments

**Do you have any other comments about using non-prescribed medicines, supplements, remedies or other therapies to manage your Long COVID symptoms?**
